# Can the ‘Deathbed Etiquette’ guide support friends and family to navigate the dying process? A qualitative study examining the views of bereaved people

**DOI:** 10.1177/26323524261488421

**Published:** 2026-09-10

**Authors:** Lara Datta Paulin, Louise Nelson, Katie Pears, Rhian Wyn Thomas, Sarah Clelland, Lesley Dunleavy, Margaret Doherty, Amy Gadoud

**Affiliations:** 1 International Observatory on End-of-Life Care, 4396 Lancaster Medical School, Health Innovation One, Lancaster University, Lancaster, UK; 2 Specialist Palliative Care Team, Lancashire Teaching Hospitals NHS Trust, Preston, UK; 3 School of Medicine, NHS England Northwest, Manchester, UK; 4 Specialist Palliative Care Team, Manchester University NHS Foundation Trust, Manchester, UK; 5 Specialist Palliative Care Team, East Cheshire NHS Trust, Macclesfield, UK; 6 The Centre for the Art of Living and Dying Well, 324073 St Mary’s University, London, UK

**Keywords:** deathbed etiquette, written resources at end of life, understanding the dying process, carer support, palliative care, end of life care, health communication, death, dying, bereavement

## Abstract

**Background:**

How people experience the death of a friend or relative is impacted by their knowledge of what to expect when someone is dying and how prepared they feel. Deathbed Etiquette is a written resource providing information about the dying process to support friends and relatives through this experience.

**Objective:**

To explore the views of bereaved people on the guide Deathbed Etiquette.

**Design:**

Qualitative study, utilising semi structured interviews.

**Methods:**

Participants were recruited through a hospice and hospital in the Northwest of England and social media between December 2023 and March 2025. Framework analysis was used to analyse the data.

**Results:**

Bereaved participants (n=22) expressed a sense of reassurance from the guide and felt that this additional written information would have been valuable. Content identified as particularly important included what the dying process physically looks like, uncertainty, what to expect after death and the needs of friends, relatives and children. The importance of an individualised approach was recognised; a generic guide may not adequately meet everybody’s needs and cultural and religious variations are not addressed in the guide. There was mixed feedback on the format of the guide, with suggestions for change. The guide should be delivered alongside a sensitive conversation with a healthcare professional and would be most beneficial when dying occurs at home. It could also be freely available in public spaces, to improve death literacy.

**Conclusion:**

Deathbed Etiquette is a useful tool to improve the experience of those supporting a friend or relative at the end of life. However, changes should be made to improve its usefulness and accessibility. An individual approach to information provision is needed and one guide cannot meet the needs of everyone. The findings can be applied more widely to other resources, however limitations in religious and cultural generalisability should be considered.

## Plain language summary

**Why was the study done?** Society finds dying a difficult topic to discuss. People may not know what to expect when a friend or relative is dying. Experts in palliative care have made a guide called ‘Deathbed Etiquette’ which includes information to help people when a friend or relative is dying. The research team wanted to find out if people that have experienced the death of a friend or relative think the guide is helpful, or not.**What did the researchers do?** The research team talked to people who have experienced the death of a friend or relative and asked them what they thought of the guide. **What did the researchers find?** The research team spoke to 22 people. The research team found that these people felt a need for more written information about the dying process and to support them through caring for their friend or relative. There were different opinions on the content and format of the guide, and it was clear that people experience the dying process differently. It is therefore a challenge to create a guide which takes all these differences into account. Participants felt that the guide would need to be introduced as part of a conversation, with a healthcare professional but they also felt it could be more widely available to the public. In addition, participants felt that there needs to be more open discussion in society about death and dying. **What do the findings mean? **This study found that the ‘Deathbed Etiquette’ guide is a useful tool for people supporting a friend or relative who is dying. It will help to develop the content of the guide and how it is used. The study has also shown that individuals want more information about the dying process and wish to reduce stigma surrounding dying.

## Introduction

Supporting a friend or relative through the dying process is a significant life event and is an experience that can have lasting implications, both positive and negative, for years to come.^1–5^ How people experience the death of a friend or relative can be impacted by their knowledge of what to expect when someone is dying and how prepared they feel.^1,2,4,6–8^ It is recognised that globally, societal knowledge of dying is reduced as deaths are more likely to occur in hospital, away from the wider family.^9^ With death being more hidden, there is a reduced awareness of what to expect when someone is dying and what dying looks like.^9^

It was thought this may have changed during the COVID-19 pandemic when, in many countries, more people died at home.^10,11^ The impact of the pandemic on the public conversation around death, however, has been mixed. Whilst awareness of death and dying is high,^10,12^ post-COVID research shows this is associated with negative attitudes such as increased fear and anxiety towards death.^9,13–15^ Furthermore, a UK survey conducted during COVID-19 found that society still doesn’t discuss death and dying enough^16^ and a Canadian study found that the public preference for dying at home was not increased during the pandemic.^17^

Information about death and dying provided verbally by healthcare professionals to those with a dying relative is valued highly.^18–20^ Relatives may also value having access to written or online information provided to them directly by healthcare professionals.^19,21–23^

In 2019, the Deathbed Etiquette guide was developed in response to this need for friends and relatives to have more written information about the process of dying.^24^ This was developed by The Centre for the Art of Dying Well at St Mary’s University in collaboration with Lancaster University Medical school and St Oswald’s Hospice, Newcastle.^24^ The guide was developed to support those people at the bedside of a dying friend or relative. Its aim is to provide reassurance and advice regarding the dying process and bedside practices. In 2020, in response to the COVID-19 pandemic, an additional version of the guide was created to support those who were unable to be at the bedside of a dying friend or relative.^25^ Research exploring the views of healthcare professionals found value in the guide and saw it playing a role in the ‘normalisation’ of death and dying.^26^

It is important that family members and friends are provided with the information they want and need to help them feel better prepared to support their dying friend or relative as well as themselves.^4,6–8,10,18,20,27^ The aim of this study was to explore the views of bereaved people on the Deathbed Etiquette guide and whether it could be a useful tool in improving the experience of friends or relatives supporting somebody at the end of life.

## Methods

### Study design

This was a qualitative interview study, conducted between April 2023 and March 2025, in which semi structured interviews were used to gain an understanding of bereaved peoples’ views on the Deathbed Etiquette guides. This study design was chosen to gain the rich data required to meet the study objectives. Both Deathbed Etiquette guides can be accessed online or distributed in paper format, on one side of paper.^24,25^ The original guide includes 15 points of information about what to expect when at the bedside of a dying friend or relative, accompanied by a digital image of a dying man surrounded by several individuals. The COVID-19 guide comprises 12 points of information which are more descriptive and relevant for those who are unable to be physically present at the bedside of a dying friend or relative. This guide also includes a digital image of a patient in bed, communicating with somebody via an electronic device.

### Context and sampling

The study was carried out in the Northwest of England. The inclusion criteria for participants were: have experienced the death of a friend or relative; aged 16+; English speaking; residence in the United Kingdom. Exclusion criteria were: bereaved within six months of recruitment; bereavement from a sudden death. There is increasing recognition that younger people value being involved in palliative care research and offer important perspectives.^28^ The age criterion of 16 years or over, as opposed to 18 years, was chosen to include the views of younger bereaved people.

Two methods of recruitment were used. The first method involved two participating organisations, a large NHS organisation and a third sector hospice organisation, inviting those whose friend or relative was cared for by that healthcare organisation to take part in the study. Potentially eligible participants were identified from bereavement databases at both participating sites and initial contact made via letter, email or telephone with an invitation to take part.

The second method of recruitment was through advertisement of the study on social media, websites and in local community centres within Greater Manchester. Potential participants responded to this via email or telephone. The aim of recruiting through these different channels was to recruit participants who had experienced bereavement in a mixture of care settings (home, hospice and hospital), who had received varying levels of specialist palliative care support and to improve representation from sub-populations (e.g. LGBTQ+ and minority ethnic groups) that are known to be under-recruited in research (NIHR, 2020).^29^

### Data collection and processing

All participants received electronic copies of both Deathbed Etiquette guides within the study invitation pack at the point of initial contact with the research team. The shortest period between receiving the guides and interview was four days, allowing participants time to pre-read the guides. Participants were also given additional time to review the guides during the interview if required.

A sample size of between 20-30 individuals was felt to be sufficient to meet the study objectives. This was determined by confidence that the interviews would produce a strong dialogue to support rich data collection, and that the study’s aims were narrow in examining responses to a specific resource. However, there was awareness that enough participants were required to reflect some of the variability of experiences when caring for someone at the end of life. These are aspects of the ‘information power’^30^ model guiding sample size in qualitative interview studies. Reviewing the data collection process of the study of healthcare professionals’ responses to Deathbed Etiquette^26^ helped to draw these conclusions. A similar sample size has also been used in other similar studies, using similar qualitative study design.^31,32^ Twenty-two semi-structured individual interviews were conducted, 21 online and one in-person which was in keeping with the wishes of participants. Both individual and focus group discussions were offered; review of the literature shows that both methods are effective in bereavement research.^33^ Nineteen participants expressed a preference for an individual interview; one preferred an online focus group and two had no preference. Focus groups were only offered in person, to allow recognition of participant distress and only in Greater Manchester, due to the locality of the researchers. Participants were recruited from across the whole of the UK; this alongside participant preference meant that all participants were individually interviewed.

The research team who conducted data collection was composed of palliative care physicians with experience of navigating conversations surrounding death and dying. There were no interactions between participants and the researchers in a clinical setting prior to the study.

When conducted online, interviews were held via a secure university-approved Microsoft Teams account. A secure dictation device was also used to record a second copy of the audio data. When conducted in-person the interview was held at the healthcare site most accessible to the participant. Audio data was recorded using the same methods as online interviews.

An interview topic guide (available as supplemental material) was utilised to conduct the interviews and provide consistency between facilitators. This included questions about overall response to the guide (positive/negative), drawbacks and limitations, how it could be implemented and in what setting(s), and responses to the COVID-19 guide. The interview topic guide was developed by clinicians specifically for this study and approved by the HRA and REC. Interviews lasted on average 45 minutes with a range of 30-62 minutes. Interviews were led by SC, LDP, LN, KP and RT, between February 2024 and March 2025.

Following the interviews, audio/video data was uploaded onto the University’s encrypted server. Interviews were anonymised and transcribed verbatim by the research team. Video recordings were then converted to audio-only for storage as per protocol.

It is recognised that individuals from marginalised groups are under recruited in research.^29^ In order to understand the diversity within our participant population, we asked participants to fill in a demographic questionnaire prior to interview. This was with the aim of providing context when exploring participants’ experiences, identifying patterns across groups, and evaluating the applicability of our results to a wider population.

### Ethical considerations

It was recognised that there was a risk of distress to bereaved participants, which was carefully considered by reviewing literature in which bereaved individuals are interviewed.^32,34^ To minimise the risk of distress, participants were interviewed at least six months following their bereavement.^32^ A robust distress protocol was also developed, should participants become upset at any point in the study. The protocol included pausing the interviews, taking participants aside, providing the option to withdraw from the study, and directing participants towards further support. It was also recognised that participants may find returning to the organisation where they were bereaved challenging and therefore alternative venues were offered.

### Data analysis

Analysis of the data followed a framework analysis approach, organising and analysing data using matrices.^35^ This involved a systematic process of sifting, charting and sorting material according to key issues and themes, following six key stages: familiarisation, coding, identifying a thematic framework, applying the framework, charting and interpretation.^36^

Data collection and analysis was an iterative process. Regular meetings between the research team were conducted throughout the study duration. This enabled new analytical codes to be added during the process and allowed identification and discussion of new themes.

SC, LDP, LN and KP re-read the transcripts and listened to the audio recordings, independently coding the transcripts. Where possible researchers coded the transcripts of interviews they had conducted to allow greater insight into the data. A combined deductive and inductive approach was used, formulating some codes based on specific questions about the guide, while other codes were developed inductively based on the data in the transcripts. A matrix was developed using these codes. LDP, LN and KP reviewed and entered data from each other’s coded transcripts to the matrix, to enable a richer understanding of the material. Key themes and comparisons across participants were inductively created through group review and discussion of the matrix with the wider research team. Triangulation back to the transcripts continued throughout the process.

## Results/findings

A total of 22 participants were interviewed, none of whom had seen or used the Deathbed Etiquette guides prior to study recruitment. During each interview, the discussion was mostly focused on the original Deathbed Etiquette guide, subsequently followed by specific questions about the COVID-19 guide. The results of the study are therefore reflective of the original guide, unless specifically highlighted otherwise.

Many participants were reflecting on more than one experience of a friend or relative dying. The term ‘friend or relative’ was agreed appropriate to indicate somebody of significance to the dying person. It was not possible to draw distinct conclusions for either of these groups as no participants reflected on the death of a friend only. Seventeen reflected on the death of a relative only and five on both a relative and a friend’s death.

We also collected data on whether the participants had experienced specialist palliative care team (SPCT) input for their friend or relative. In the UK, this generally consists of support from a multi-disciplinary team with specialist experience in palliative care, which can be provided across multiple care settings. Referral would usually be triggered by complexity of symptoms or holistic support needs. Fifteen out of twenty two participants had experienced SPCT involvement during the dying process of their friend or relative. Further demographics can be seen in Table 1.

**Table 1.** Participant Demographics.

Variable	Number of participants (n=22)
**Age (years)**	
25-34	1
35-49	5
50-64	10
65-79	5
Missing	1
**Gender**	
Female	15
Male	7
**Ethnicity**	
White - English, Welsh, Scottish, Northern Irish or British.	18
White - Irish	2
Any other white background	1
Mixed or multiple ethnic groups - White and Asian	1
**Religion**	
Christian	12
No religion	8
Buddhist	1
Prefer not to say	1
**Sexual Orientation**	
Straight/heterosexual	18
Gay or Lesbian	2
Bisexual	1
Prefer not to say	1
**Diagnosis leading to death**	
Cancer	12
Non-malignant disease	10

Three broad themes emerged from the data: what information is helpful in a written guide for people supporting someone at the end of life; design and format of the guides; and delivery of the guides.

### Theme 1 – What information is helpful in a written guide for people supporting someone at the end of life?

There was variation in how much information participants had been given during their experiences of the dying process prior to the study. This was influenced in part by whether there was input from a SPCT – in these cases participants generally reported better information provision. However, information was often provided verbally and did not include specific details about what the dying process looks like.

Participants therefore had an overall positive response to the guide as it provided a sense of reassurance. It also provided information that they had not otherwise been given (either verbally or through other resources) or information that they had needed to seek out independently.

*“This is normal. These things are going to happen. You’re not having a weird experience. Well, you are having a weird experience but it’s helpful to normalise it.” P16, Female*

Participants felt strongly that in addition to the information they had received during their experience, any written information about the dying process would have been valuable.

*“I hadn’t had anything like that when my husband died and I think it would be quite useful to have had something to just give those pointers” P1, Female*

Those whose friend or relative had received SPCT input still valued the guide, however highlighted that in scenarios where this support is not available or accessed, the need would be even greater.

Participants felt that information about what the dying process physically looks like was particularly important in the guide.

*“Something to look at that just tells you sort of what may happen or what may happen next, and that allays your fears because you are listening to sounds and breathing and things that you haven't heard of or experienced before.” P7, Female*

Participants also expressed a wish for information around the uncertainty of the dying process and what to expect in the immediate moments after death, neither of which are provided in the current guide.

*“And there's something about, some people die quick and easy, and some people take forever...and there's something in my head about the enormous differences in the way, in. I’m not sure ‘way’ is the right word, way, how people die. And it's just so different.” P9, Male*

*“Right up to the moment she died, there was someone else with me. And then she wasn't there. And then it's like it's just me now and that was almost the hardest thing, because when there's somebody else alive, even though they're dying, you're there for them. But when they've gone and you know, it's all those cliches about a light going out and everything. It's just like this horrible silence and you've got a dead body in your front room” P2, Female*

It was also suggested that including information to signpost readers to other sources of support or information would be beneficial. This included the encouragement to seek further information from healthcare professionals, as is mentioned in the COVID-19 version of Deathbed Etiquette.

Recognition of the needs of friends and relatives, in addition to those of the dying person, was felt to be particularly important and participants valued this within the guide.

*“But yeah, look after yourself. Make sure you've got the support that you need around you. That is really, really important. That definitely stood out, and it's definitely important. You know, and you have to eat. And like I say, for X it would have been like 2 weeks really. And you can't exist on no sleep, no food, no time for yourself, no fresh air. For two weeks, you cannot sit in a room with somebody for two weeks solid. You know you need to. You need to do the best that you can for yourself as well.” P3, Female*

Several participants felt that children are sheltered from death and dying and that this can have a negative impact on their understanding of death as a part of life, and on their acceptance of the death itself. Although the guide includes a suggestion for allowing children to visit, some participants also expressed a wish for more information about how to talk to children about dying and how to support children when visiting a dying person. Whilst there was recognition from some participants that the decision to include children in the dying process is very personal to a situation and family, only one suggested altering this section of the guide to be less prescriptive with the addition of *“if appropriate” (P7, Female)*. Many others felt favorably towards inclusion of this in the guide.

Importantly, participants also highlighted that cultural and religious variations are not addressed in the guide and that in relation to these, information needs may vary.

*“It’s for a very white, you know, possibly either Christian, non-Christian, but it's not, I don’t think it says to me that its very inclusive of other faiths, other religions, other cultures” P15, Female*

Suggestions as to how the guides could be changed were broad and there were numerous different ideas about what would be helpful to add. Participants also had many suggestions regarding additional guides/sources of information that could be developed for further support. For example, resources with information about deaths in specific circumstances and resources explaining what to do in the moments after death. Given the diversity in participants’ experiences of death, it was acknowledged by many that some aspects of the Deathbed Etiquette guides would not apply to all deaths or all individuals. Specific situations where the guides were felt to be less applicable included sudden deaths, deaths due to assisted dying/euthanasia, deaths of children and when there is significant emotional or family complexity.

Many participants expressed the importance of having an individualised approach to information provision and that for some, a generic written guide may not adequately meet their needs.

*“Dying is a different experience for everyone, not everything will be relevant. I think it’s just very difficult to have one leaflet to cover every, you know, every circumstance. That’s the difficulty isn't it?” P14, Female*

### Theme 2 – Design and format of the guides

Participants gave a wide range of detailed opinions on the design and format of the guides. This included opinions on the title, imagery, colour and font used in the Deathbed Etiquette guides.

Most participants valued the direct nature of the ‘deathbed’ part of the title. They felt the clarity this provided was valuable. Only two participants felt negatively towards the ‘deathbed’ part of the title. One due to the directness of the term and another identified the term ‘deathbed’ as having negative connotations in their culture.

The ‘etiquette’ part of the title was perceived more negatively by the majority of participants. This opinion was expressed more strongly by female rather than male participants. For those who disliked the use of the word ‘etiquette’, the rationale was consistently due to its unfamiliarity and formality, and the implication of a correct way to behave when a friend or relative is dying. There was a concern that feelings of behaving incorrectly could lead to guilt. Participants also highly valued the acknowledgment of feelings of guilt in the COVID-19 guide.

*“But I don't like the word etiquette. It feels quite formal and links to what I've said actually about like there's no rules basically. The rules should be the rules that you should decide, so I guess is the word etiquette a word that people connect with being posh and silver forks and China plates rather than umm… I guess I don't even know what, I don't know what word I'm I would think that is more in tune, but it feels quite it feels like quite a formal word for something that's much more informal” P6, Female*

Across age groups, there was no variation in opinions on either aspect of the title.

Participants expressed a wide range of opinions regarding the images used in the guides, some of which were conflicting. Male participants placed lower importance on the images, with some suggesting an image wasn’t needed. Criticism of the images included perceived immaturity due to the cartoon design, whereas other participants valued the use of a cartoon image rather than a photograph. Some felt that a symbol would be more appropriate. A consistent opinion among participants was that it was important that the images accurately represented dying. They valued the representation of different generations in the images however, they didn’t feel the images reflected diversity in ethnicity, culture, or setting.

*“I think the picture needs to change. I think it needs to be a bit more inclusive, shall we say of actually what death, how death and what death can look like. Now how you do that and how you incorporate that…but maybe it's not focusing on your stereotypical, of what one might think when we think of death of an older person dying with young family around looking sad. I think it probably needs to be a bit more inclusive of how you know what death can look like for lots of other people as well.” P15, Female*

Regarding the format of the guide, there was again variability in opinions across all demographic groups. Overall, participants valued information being provided in a concise manner, with bold titles and more detail below these titles. The difference in format of the original and COVID-19 guides allowed participants to compare these and discuss what they valued from each guide. When discussing the COVID-19 guide, participants liked having the option to read ‘headlines’ followed by more detailed text and felt this would be a valuable addition to the original guide. Several participants suggested combining the original and COVID-19 guides. Suggestions included changing the guides from a single page into a different format, such as a small booklet, to make it easier to digest the information. Accessibility of the guides was also highlighted by participants with recognition of the limitations for those not fluent in written English, those with sight difficulties, and those unable to read.

### Theme 3 – Delivery of the guides

There were two main ways that participants felt that the Deathbed Etiquette guide should be provided. Participants felt the guide should be provided as part of a sensitive conversation with a healthcare professional, such as a GP or SPCT professional (medical or nursing). Participants also believed the guide could be available for the public, for example in spaces such as GP surgeries, pharmacies and hospital visiting areas.

*“I'm not sure that I would have liked to have just been handed it and you know, and left left to it. I think it does need to be and sort of handled with sensitivity and with a bit of context” P1, Female*

A consistent opinion was that the healthcare professional delivering this information was familiar to the friend or relative and had the required knowledge and communication skills to have the conversation. Those participants who had received SPCT input in their experience, felt these guides could be delivered by non-SPCT or SPCT professionals alike. Those with no SPCT input did not specifically reference the SPCT. Some participants felt there was additional value in having repeated conversations regarding the guide when it is introduced.

Participants felt there would be value in people, including healthcare professionals, having the opportunity to pick up the guide in public spaces. This could be read at any time including outside of the context of a friend or relative dying. Improving accessibility and death literacy was felt to be important.

It was felt that the guide would be valuable for friends or relatives of people dying in different settings but particularly when their friend or relative was dying at home, due to the increased isolation and uncertainty associated with this. This opinion was not influenced by the setting of participants’ personal experience. Participants felt that the use of this guide in a hospice setting was less valuable as the information would already be provided by hospice staff, an opinion expressed by participants irrespective of the setting in which their friend or relative died. Participants felt that while the guides would still be useful in a hospital setting, it would be difficult to follow all the advice included in the guide, such as making the bedspace personalised.

The timing of provision of the Deathbed Etiquette guide was discussed. Overall participants felt that this information should be provided when dying is recognised as a reasonable possibility. More specific suggestions of when to provide the guides included: when providing anticipatory sub-cutaneous medications for symptom control at end of life; when active treatment is stopped; or at initial contact with the team supporting the patient, such as on admission to the hospice or hospital, or at the first meeting with a district nurse or specialist palliative care nurse.

## Discussion

Bereaved people believe that there is a need for greater information when somebody is at the bedside of a dying friend or relative than that currently being provided. They value this information in written form, and guides such as Deathbed Etiquette or similar could be highly beneficial. However, considerations need to be made about the content, design and delivery of such guides, and changes to the current Deathbed Etiquette guide would improve its accessibility and its use.

### What information is helpful in a written guide for people supporting someone at the end of life

Our findings confirm that friends and relatives have a desire for information provision regarding the dying process and after death.^20^ Our study confirms the additional value that written information can provide, which previous research has found helps relatives confirm and clarify what they are experiencing.^23^ Our study also supports existing research showing that written information for relatives provided in palliative care settings should include signposting to other information sources.^23,37^ However, our study adds more specific understanding of the needs regarding the content of written information provided for friends and relatives. It is important to friends and relatives that information includes recognition of individuality of experience and what to do immediately after death. The usefulness of the Deathbed Etiquette guides would be improved by the addition of these details as would other similar resources. Beyond recognition of individuality, the importance of written information and its content being applicable to different cultures has been highlighted by our study, which is in keeping with the views of the previous healthcare professionals study.^26^ The importance of this cannot be underestimated with existing research highlighting the value of recognising cultural differences in palliative care brochures and that it is essential that different cultural groups are consulted when developing such resources.^38^ It has been suggested that written information may be of less value in specific cultures.^38^ This has implications for the value and accessibility of such resources for different cultural groups and therefore their ‘health or death literacy’.^38^ Further research to seek the views of ethnically diverse communities would add value to the Deathbed Etiquette guides.

Research has shown that in the UK and internationally there is public support for inclusion of grief education in schools, and it is felt that this, particularly in secondary education, may improve understanding of death and dying in society.^16,39^ Our study showed that it is important to friends and relatives that resources like Deathbed Etiquette include consideration of children’s needs. Participants valued this as they felt it would result in increased involvement of children when a friend or relative is dying, with the potential to therefore improve wider death literacy. Participants identified a need for improved societal knowledge of death, in keeping with existing research.^16,39^

### Design and format

Our study showed that the design and format of the Deathbed Etiquette guides was very important to friends and relatives, with some indication this might be more important to the female participants.

Previous research that explored the views of healthcare professionals on the Deathbed Etiquette guide found they valued the short sentences and simple language, placing high importance on the language used.^26^ Other research has confirmed that families and patients also value the use of clear, concise and accessible language in Advance Care Planning pamphlets.^23^ Our findings build on this previous work by confirming similar opinions amongst friends and relatives regarding the language used in the Deathbed Etiquette guides and the importance of this. This shows that careful consideration should be given to the language used in such guides, which is particularly important when considering the title. In both the healthcare professional study and our study, participants had strong views on the title ‘Deathbed Etiquette’.^26^ In the healthcare professionals’ study, the term ‘deathbed’ was felt to be too direct.^26^ In contrast, in our study, bereaved people responded positively to the word ‘deathbed’. This perhaps identifies the gap between friends and relatives’ wishes for direct communication and what professionals feel they are comfortable with. This may contribute to the sense of stigma around death and dying if professionals feel it is too direct to use words such as ‘deathbed’ in the context of a societal desire for more discussion around death and dying.^16^ Both healthcare professionals, and friends and relatives, disliked the use of the word ‘etiquette’ due to the implication of there being rules to follow.^26^ Given the value placed on the language used by participants, the title of the guides should be changed, which may improve the use of the Deathbed Etiquette guides. The principle of friends and relatives valuing direct and clear language such as ‘deathbed’ can be applied to written resources more widely in palliative care. However, our finding that ‘deathbed’ has negative connotations in some cultures emphasises the need for diverse cultural opinions when developing a new title.

Our finding that the images used in the Deathbed Etiquette guides should more accurately represent dying in terms of ethnicity, culture and setting is supported by existing research.^38^ It is recognised that it is not only important to consider different cultures in images used in written resources but also in the language, text structure and symbols.^38^ This again highlights the value that further research beyond our study, seeking the views of participants from different cultures, would add. Healthcare professionals found the images used in the Deathbed Etiquette guides to be too hospital focussed, supporting our findings that the setting depicted in the images is important.^26^ The images used in the Deathbed Etiquette guides should be reviewed to more accurately reflect dying.

### Delivery

It is widely recognised that written resources used in end-of-life care are best provided with a conversation from healthcare professionals.^19,21–23,26,37,38,40^ Our findings support this. Existing research has explored family and patient opinion, as well as bereavement outcomes. This has shown that a conversation alongside written information is most valuable and reduces distress.^19,21–23,26,37,40^ Participants in our study provided a similar rationale for their value of conversation when receiving the guide. Our findings placed importance on the healthcare professional delivering the Deathbed Etiquette guide having the correct knowledge and skills, which is in keeping with previous findings.^22,26^ There is uncertainty about the correct timing of provision of written information and associated conversations, with some work showing that relatives protect family from receiving information about death and dying but other research showing that being proactive might be beneficial.^19,23,26^ This need to recognise individuality when deciding on timing of delivery of information was confirmed in our study.

Our participants also saw value in the Deathbed Etiquette guide being more widely available in public spaces. This contradicted the views of healthcare professionals and again this perhaps indicates a paternalistic approach by professionals when discussing death and dying.^26^ Existing research confirms our participants’ views of the value in having these resources available in public spaces.^23^ This suggests the public have a desire for information regarding death and dying to be widely available and we should support them with having access to this in the form of the Deathbed Etiquette guide and other resources.

### Implications

Guides like Deathbed Etiquette should include information about what dying looks like physically and what happens after death. They should also acknowledge uncertainty in the dying process and recognise the individual nature of dying. One guide cannot suit all individual preferences or circumstances, and this needs to be acknowledged within the guide. It is important that religious, ethnic, and cultural differences are recognised. Even with the low religious and ethnic diversity in this study, preference differences were identified. Information about helping children to navigate the process of a friend or relative dying should be considered in the development of these guides, either within the guide itself, or in a targeted resource.

The title requires review and ‘etiquette’ should be removed. Any image used needs to depict an accurate representation of dying in terms of culture, ethnicity, age, and setting. A separate guide for those unable to be at the bedside of a friend or relative is not necessarily required, given that many points are jointly applicable. However, it would be important to ensure reference within the original guide that some people are unable to be at the bedside.

Guides, such as Deathbed Etiquette, should be delivered by a healthcare professional with the appropriate knowledge and skills, as part of a sensitive conversation. This would be of even more importance when there is no SPCT input. It would be most useful to deliver such a guide at the point where dying is recognised as a reasonable possibility. Guides like Deathbed Etiquette could also be available more widely as a public resource. The Deathbed Etiquette guide could be valuable in all settings but is most useful when a friend or relative is dying at home. In its current form, it is more applicable to those who are white, British and either of a Christian faith or atheist.

### Limitations

Recruitment for the study was predominantly through voluntary responses to advertisement via bereavement and death literacy social media channels. This may add some bias to the opinions and views expressed by the participants. In addition, 59% of the study participants had a professional link to either healthcare or bereavement; this was, however, widely varied in role and setting. By nature of the study, all participants had previously experienced the death of a friend or relative. It is uncertain therefore whether the results of the study fully reflect the likely response to the guide from an individual with no previous experience of somebody close to them dying. In addition, no participants had experience of using the Deathbed Etiquette guide when their friend or relative was dying and therefore all views were retrospective. Recruitment of men, although initially poor, was improved with targeted recruitment. This resulted in gender diversity which is in keeping with other, similar studies. There was, however, a lack of ethnic and religious diversity in the study as discussed above.

## Conclusion

Our study of bereaved people confirms the value of the Deathbed Etiquette guide in providing otherwise unknown information about the dying process. However, changes should be made to the guide to improve its usefulness and accessibility. Changes should include the addition of recognition of individuality of experience and what to expect in the immediate moments after death. The addition of more information regarding the needs of children should be considered. The images should be updated to more accurately represent dying and diversity in culture, ethnicity and place of death. The title should be reviewed, in particular the use of the word ‘etiquette’. The views from ethnically diverse communities should be understood in future research. Our findings support previous research that written resources, such as the Deathbed Etiquette guides, should be provided with a sensitive conversation by a healthcare professional with the appropriate skills and knowledge.^19,21–23,26,37,38,40^ However, there is uncertainty in our participants’ views and previous work about the correct timing of this. This highlights the importance of approaching information provision in end-of-life care in an individualised manner. Given the individuality of experience, it should be recognised that one guide cannot meet the needs of everyone. Our participants felt the Deathbed Etiquette guide would also be helpful if available in public spaces indicating an ongoing appetite from the public to have more information about dying easily available to them. The findings of our study can be applied more widely to other written resources used in end-of-life care, although limitations in terms of religious and cultural generalisability should be considered.

## Supplemental material

Supplemental material - Can the ‘Deathbed Etiquette’ guide support friends and family to navigate the dying process? A qualitative study examining the views of bereaved people Supplemental material for Can the ‘Deathbed Etiquette’ guide support friends and family to navigate the dying process? A qualitative study examining the views of bereaved people by Lara Datta Paulin, Louise Nelson, Katie Pears, Rhian Wyn Thomas, Sarah Clelland, Lesley Dunleavy, Margaret Doherty, and Amy Gadoud in Palliative Care and Social Practice.

Supplemental material - Can the ‘Deathbed Etiquette’ guide support friends and family to navigate the dying process? A qualitative study examining the views of bereaved people Supplemental material for Can the ‘Deathbed Etiquette’ guide support friends and family to navigate the dying process? A qualitative study examining the views of bereaved people by Lara Datta Paulin, Louise Nelson, Katie Pears, Rhian Wyn Thomas, Sarah Clelland, Lesley Dunleavy, Margaret Doherty, and Amy Gadoud in Palliative Care and Social Practice.

## Data Availability

The datasets generated during and/or analysed during the current study are available from the authors on request. For access to any underlying research materials please email: a.gadoud@lancaster.ac.uk.
