# Supplementary material for: Can the ‘Deathbed Etiquette’ guide support friends and family to navigate the dying process? A qualitative study examining the views of bereaved people
Source: Palliat Care Soc Pract. 2026 Sep 10;20:26323524261488421. doi: 10.1177/26323524261488421 (PMC13562848; doi:10.1177/26323524261488421)
Supplement: Supplementary file 1 [file sj-pdf-1-pcr-10.1177_26323524261488421.pdf]

Interview Topic Guide

Exploring the views of bereaved people on using the guide to Deathbed Etiquette in end of life care

<u>Pre-recording:</u>

- Make sure you have copies of the guides to look at/refer to.
- There should be two people running a group interview: one to facilitate the group and one to take notes and/or support in case of any problems. Individual interviews can be conducted by one researcher.
- A group interview needs to have at least three participants. If fewer than three participants attend a face to face group interview, then gain participant agreement to split off into individual face to face interviews.
- Check everyone has consented to take part in the study. For participants consented before the interview day, consent will be confirmed verbally again. Explain (where relevant) that we expect an individual interview to last approximately 1 hour and a group interview to last 1-2 hours.
- **Group interview ground rules:**
    - Everyone's perspective is equally valid.
    - Different views are expected and encouraged.
    - Negative as well as positive comments about the guide are just as important to the evaluation process.
    - Please avoid using your name or your friend or relative's name.
    - Please avoid naming specific organisations~~ o~~ or individuals involved during the care of your friend or relative.
    - All discussions taking place in the group interview should remain confidential and should not be discussed outside that group interview.
    - There are some limits to confidentiality: if what is said makes the research team think that you, or someone else, is at significant risk of harm, they will have to break confidentiality and report this to the research lead and possibly other relevant parties. If possible, you will be informed about this.

- For online interviews: using Microsoft Teams is secure and approved by the University of Lancaster, however, the internet cannot be completely guaranteed as a secure means of communication. Participants to confirm they are happy to proceed.
- For online interviews: check participants can use hand function to indicate they want to speak.
- Check if anyone has any questions about the study or consent form beforehand.

Switch recording on (including second recorder if using), state the date of interview and name(s) of participant.

<u>Opening questions</u>

Can you introduce yourself?

Have you had any prior experience of the guide? What do you know about the guide already?

Give group opportunity to lead the discussion. May be useful to allow a few minutes to look at the guide if they haven't had chance yet.

<u>Whether the guide is helpful and why</u>

For those who were already aware of the guide, can you describe what your initial thoughts were upon coming across it for the first time?

For those who have only just read through the guide, perhaps you'd like to talk about your initial reaction to it? What are your first thoughts?

Are there some parts of the deathbed etiquette guide that stand out to you as particularly important? Why?

Those who have not used the guide - do you feel you were given this information in another way, and if so, how?

How do you imagine the guide might help support patients and families?

<u>Potential drawbacks or limitations to using the guide</u>

Is there anything you don't like about the guide?

Is there anything you think is missing/ should be added?

Are there any other ways you would change the guide?

Are there any situations where you don't think using the guide would be helpful?

Can you see any drawbacks or limitations to using the guide?

<u>When the guide would be best introduced and how</u>

How do you think the guide could be used for those looking after someone at the end of life?

Do you think it could be given to everyone, left by bed, or only given to select people and/or as part of a conversation?

At what point would it be appropriate to introduce the guide?

Who do you think would be best to introduce the guide?

What questions might you want to ask someone who was giving you the guide?

<u>Helpfulness of the guide in a hospice, hospital and home setting</u>

Do you think the guide would be more or less helpful in any particular places (i.e. for people being cared for at home or in a hospice or hospital)?

<u>Helpfulness of the COVID-19 guide when it is not possible to be at the bedside of a friend or relative who is dying</u>

The guide was revisited in light of the COVID pandemic. Having read this version of the guide, what are your thoughts on it?

For those who have used this guide, can you describe your experiences of doing so?

Those who have not used the guide, how helpful do you think it might be for those who are unable to be at the bedside of a friend or relative who is dying?

What might you want to change or add to this?

Do you have any concerns about using it?

<u>Closing questions</u>

Are there any further thoughts you'd like to share about the guide?
