# Supplementary material for: Can the ‘Deathbed Etiquette’ guide support friends and family to navigate the dying process? A qualitative study examining the views of bereaved people
Source: Palliat Care Soc Pract. 2026 Sep 10;20:26323524261488421. doi: 10.1177/26323524261488421 (PMC13562848; doi:10.1177/26323524261488421)
Supplement: Supplementary file 2 [file sj-pdf-2-pcr-10.1177_26323524261488421.pdf]

Distress Protocol

Exploring the views of bereaved people on using the guide to Deathbed Etiquette in end of life care

Participant shows signs of distress:

- Stating that they are distressed or upset
- Showing physical signs such as uncontrolled crying, incoherence, shaking

Stage 1 Response:

- Stop the interview
- Offer to take participant aside if in a group
- Ask how they are feeling:
  "What are you feeling right now?"
  "Do you feel able to go on about your day after this interview?"

Review:

- If the participant feels happy to continue then resume the interview
- If the participant would like to pause before continuing, take a break or offer to reschedule the interview
- If they are taking part in a group interview, offer the option to take part in an interview at a different time
- If the participant is unable to carry on, go to Stage 2

Stage 2 Response:

- With permission, withdraw the participant from the study
- Signpost participant to further resources (e.g. wellbeing support at participating site and/or third sector bereavement support)

Participant raising concerns about an aspect of their friend or relative's care:

Stage 1 Response:

- Pause the interview and acknowledge concerns raised by the individual
- If in a group interview, explain that you would prefer to discuss this separately from the group both for confidentiality reasons, and so that you can identify if any further support is needed
- Ask whether they would like to stop the interview at that point or continue

Review:

- If the participant feels happy to continue, then resume the interview, reassuring the participant that you will speak to them further about their concerns after the interview
- If the participant would like to pause before continuing, take a break or offer to reschedule the interview. If they are taking part in a group interview, offer the option to take part in an interview at a different time

Stage 2 Response:

- Acknowledge the concerns raised separately with the participant ideally immediately after the interview is completed, or when they elect to stop
- Direct the participant to appropriate help such as PALS (or equivalent) at the relevant healthcare organisation
